# Responses of biomass allocation across two vegetation types to climate fluctuations in the northern Qinghai–Tibet Plateau

**DOI:** 10.1002/ece3.5194

**Published:** 2019-05-06

**Authors:** Licong Dai, Xun Ke, Xiaowei Guo, Yangong Du, Fawei Zhang, Yikang Li, Qian Li, Li Lin, Cuoji Peng, Kai Shu, Guangmin Cao

**Affiliations:** ^1^ Key Laboratory of Adaptation and Evolution of Plateau Botany, Northwest Institute of Plateau Biology Chinese Academy of Science Xining China; ^2^ University of Chinese Academy of Science Beijing China; ^3^ College of Life Sciences Luoyang Normal University Luoyang China

**Keywords:** climate variables, interannual variation, plant biomass, Qinghai–Tibet Plateau, vegetation types

## Abstract

The Qinghai–Tibet Plateau (QTP) is particularly sensitive to global climate change, especially to elevated temperatures, when compared with other ecosystems. However, few studies use long‐term field measurements to explore the interannual variations in plant biomass under climate fluctuations. Here, we examine the interannual variations of plant biomass within two vegetation types (alpine meadow and alpine shrub) during 2008–2017 and their relationships with climate variables. The following results were obtained. The aboveground biomass (AGB) and belowground biomass (BGB) response differently to climate fluctuations, the AGB in KPM was dominated by mean annual precipitation (MAP), whereas the AGB in PFS was controlled by mean annual air temperature (MAT). However, the BGB of both KPM and PFS was only weakly affected by climate variables, suggesting that the BGB in alpine ecosystems may remain as a stable carbon stock even under future global climate change. Furthermore, the AGB in PFS was significantly higher than KPM, while the BGB and R/S in KPM were significantly higher than PFS, reflecting the KPM be more likely to allocate more photosynthates to roots. Interestingly, the proportion of 0–10 cm root biomass increased in KPM and PFS, whereas the other proportions both decreased, reflecting a shift in biomass toward the surface layer. Our results could provide a new sight for the prediction how alpine ecosystem response to future climate change.

## 1 INTRODUCTION

Grasslands are the most widely distributed ecosystem type, and make a great contribution to terrestrial ecosystem carbon stocks (Ma, Yang, He, Zeng, & Fang, 2008; Sun, Cheng, & Li, 2013). Some previous studies have shown that grasslands account for almost 10% of global carbon stocks, and play a vital role in global carbon assessment (Scurlock & Hall, 1998). Therefore, understanding the temporal dynamics of biomass under climate fluctuates is of great importance in elucidating the response mechanisms of alpine plant to climate change in the future (Roa‐Fuentes, Campo, & Parra‐Tabla, 2012).

In recent years, a great number of studies have been documented regarding the response of alpine ecosystems to climate change, such as precipitation gradients (Roa‐Fuentes et al., 2012; Zhou, Fei, Sherry, & Luo, 2012), warming, and drought (Bloor, Pichon, Falcimagne, Leadley, & Soussana, 2010; Day, Ruhland, & Xiong, 2008; Xu, Peng, Wu, & Han, 2010), as well as other environmental factors (Hamelin, Gagnon, & Truax, 2015; Sun et al., 2013). However, most previous studies were just based on experiments or observed climatic gradients; few studies have directly explored the response of the alpine ecosystem to climate change in natural ecosystems. It should be noted that the warming experiments only simulated the response of plants to warming and did not represent the real‐world climate warming scenario; previous study has observed that the artificial warming experiments usual systematically underestimate the effect of real‐world climate warming on ecosystems (Wolkovich et al., 2012). Therefore, it is necessary to use long‐term observation data to better predict the response mechanism of alpine ecosystems to climate change. Furthermore, most previous studies have only focused on the response of alpine meadows to climate change based on long‐term observation data, while changes in other vegetation types, such as alpine shrubs, are still poorly understood (Nie, Feng, Yang, Li, & Zhou, 2017; Nie et al., 2019), especially considering that the response to climate change varies significantly between vegetation types. Thus, it is crucial to combine the study of different vegetation types to have a better understanding of the responses of alpine ecosystems to climatic changes in the future. In addition to air temperature, the surface soil temperature was also found to be increased due to warmer climates, especially in the top 10 cm layer (Wu et al., 2014). Such change in soil temperature may substantial affect the soil water content and soil nutrients availability; thus, the plant may change their vertical distributions to response to the altered the climate change, many studies found a shift in root distribution moved toward the deeper or surface soil layer owing to warming (Wu et al., 2014; Xu et al., 2010). However, very few studies have focused on these influences in alpine grasslands.

The Qinghai–Tibet Plateau (QTP) is known as the highest and largest plateau on earth, and also the principal area of alpine meadow and alpine grassland, covering almost 46% of the plateau (Yang, Fang, Ma, Guo, & Mohammat, 2010), which make great contribution in global carbon cycle and carbon pool. However, this ecosystem is particularly sensitive to global change compared with other ecosystems, especially elevated temperature. In the past decades, the Tibetan Plateau has experienced a dramatic rise in air temperature during the last 50 years, almost at a double rate that of the global average (Dong, Jiang, Zheng, & Zhang, 2012), which substantial alter the allocation pattern of biomass. According to the functional equilibrium hypothesis (optimal partitioning), plant biomass allocation is size‐independent, which suggests that plants will develop larger root systems if soil resources are limiting and will proportionally allocate more resources to stems and leaves if an aboveground resource, such as light, is limiting (Sun & Wang, 2016; Sun et al., 2014). Moreover, the belowground biomass almost accounts for 80% in alpine grassland, whereas most previous researchers have used the R/S to assess belowground biomass, because of the difficulty in obtaining belowground biomass data in the harsh environment of the QTP, but this approach is associated with larger errors owing to differences in root sampling and methodological problems, and the R/S ratio is often overestimated because of the influence of grazing. The combination of these factors ultimately leads to inaccuracies when assessing root biomass or carbon stocks (Jackson et al., 1996). Therefore, it is necessary to obtain long‐term measure biomass data to evaluate the carbon sink of northern Tibet and to examine its relationship with climate factors. Meanwhile, the area's low population density, together with relatively fewer human activities in this region, provide a unique location for studying the temporal distribution patterns of biomass in different vegetation types and their relationships with climate variables.

The major objective of the present study was to discuss the temporal distributions of biomass within two vegetation types and their relationships with climate factors. Specifically, this study has the following aims: (a) examine the interannual variations of plant biomass within two vegetation types during the period 2008–2017; (b) explore the relationships between climate variables and biomass among the two vegetation types; and (c) examine the vertical distribution of root biomass under climate fluctuations.

## 2 MATERIALS AND METHODS

### 2.1 Study area

The study was conducted at Haibei National Field Research Station on the northeast Tibetan Plateau, which has a typical plateau continental monsoon climate, average elevation is 3,200 m, and mean annual air temperature is 1.7℃. Average annual precipitation is approximately 580 mm and falls mainly during the growing season (i.e., from May to September). The summer is warm and rainy with an average temperature of 9.8℃; the winter is cold and dry with an average temperature of −14.8℃. And the seasonally frozen ground is well developed in this region. The two vegetation types at our sites are *Kobresia pygmaea* meadow (KPM) and *Potentilla fruticosa* shrubs (PFS). There was a thick Mattic Epipedon (dense organic‐rich turf) in KPM. The dominant species are *Kobresia pygmaea* and *Kobresia humilis* in *Kobresia pygmaea* meadow, and *Potentilla fruticosa* and *Koeleria cristata* in *Potentilla fruticosa* shrubs. Details of basic environmental characteristics of the two vegetation types are described in Table 1. The two study sites were located in fence‐protected areas; thus, there was little disturbance from human or grazing activities.

**Table 1:** The soil properties of different soil layers in the two vegetation types

		Soil property				
Vegetation types	Soil depth (cm)	SOM (g/kg)	AP (mg/kg)	AK (mg/kg)	AN (mg/kg)	TN (g/kg)
KPM	0–10	149.01 ± 3.04	10.94 ± 2.43	261.84 ± 14.63	19.35 ± 1.83	6.76 ± 0.42
	10–20	101.57 ± 2.62	7.66 ± 1.93	150.64 ± 11.37	15.26 ± 1.60	5.05 ± 0.29
	20–30	71.37 ± 2.78	4.57 ± 1.13	121.36 ± 9.25	13.38 ± 1.66	3.64 ± 0.24
	30–40	48.59 ± 3.31	2.30 ± 0.78	94.52 ± 11.61	9.84 ± 2.22	2.37 ± 0.27
PFS	0–10	138.61 ± 6.98	8.46 ± 1.86	292.78 ± 22.43	24.60 ± 2.85	6.22 ± 0.53
	10–20	112.21 ± 5.69	5.82 ± 1.49	156.20 ± 12.92	18.78 ± 1.81	4.97 ± 0.49
	20–30	91.27 ± 5.30	4.41 ± 1.21	99.69 ± 6.09	15.33 ± 1.87	4.17 ± 0.39
	30–40	73.61 ± 4.29	3.35 ± 1.19	91.27 ± 10.03	13.14 ± 1.61	3.51 ± 0.34

Note

PFS represents *Potentilla fruticosa* shrubs, KPM represents *Kobresia pygmaea* meadow, the same below.

### 2.2 Data collection

The belowground biomass (BGB) and aboveground biomass (AGB) were measured monthly during growing season (i.e., from May to September) among two vegetation types from 2008 to 2017. The AGB was measured using the standard harvesting method in 10 randomly selected quadrats (50 cm × 50 cm), in plots comprising the two vegetation types (100 m × 10 m), three plant functional groups were measured in each quadrat: sedges, grasses, and forbs. The 10 randomly selected quadrats were constrained to have horizontal spacings of 10 m. The BGB was sampled from soil cores (diameter 7 cm) extracted from each quadrat (50 cm × 50 cm) at depths of 0–10 cm, 10–20 cm, 20–30 cm, 30–40 cm on the basis that over 93% root biomass is concentrated in the top 40 cm of soil (Cao, Du, Wang, Wang, & Liang, 2007), with five reduplications, then cleaning the root and remove the soil particles. Finally, both AGB and BGB samples were oven‐dried at 65°C to a constant weight. In this study, the root‐to‐shoot ratio (R/S) was calculated as the ratio of BGB to AGB.

Given the two sites are approximately 5 km from meteorological station; thus, the climatic data mean annual air temperature (MAT) and mean annual precipitation (MAP) were collected from the meteorological station from 2008 to 2017.

### 2.3 Data analysis

First, the median values of AGB, BGB, and R/S ratio in the two vegetation types were calculated, and all data were tested for normality. One‐way ANOVA was used to examine the differences between AGB, BGB, and R/S ratio among the three vegetation types. Then, ordinary least squares (OLS) regression analysis and two‐way ANOVA were applied to examine the effect of climate variables on biomass among two vegetation types. The vertical distribution of roots was assumed to be characterized by an asymptotic function, following Gale and Grigal (1987), as follows:

$$Y=1-\beta^{d}$$

where *Y* is the cumulative percentage of root biomass from the soil surface to deep soil, *d* (cm) is the depth of soil, and *β* is the estimated parameter. The values of *β* represent the allocation pattern of belowground root biomass, and range from 0 to 1, where 1 indicates that all root biomass is located in deep soil, while 0 indicates that all root biomass is at the surface. All data analysis was conducted using the software package R (R Development Core Team, 2006), and all figures were plotted using Origin 9.0 (OriginLab).

## 3 RESULTS

### 3.1 Interannual variations of climate factors

**Figure 1:**
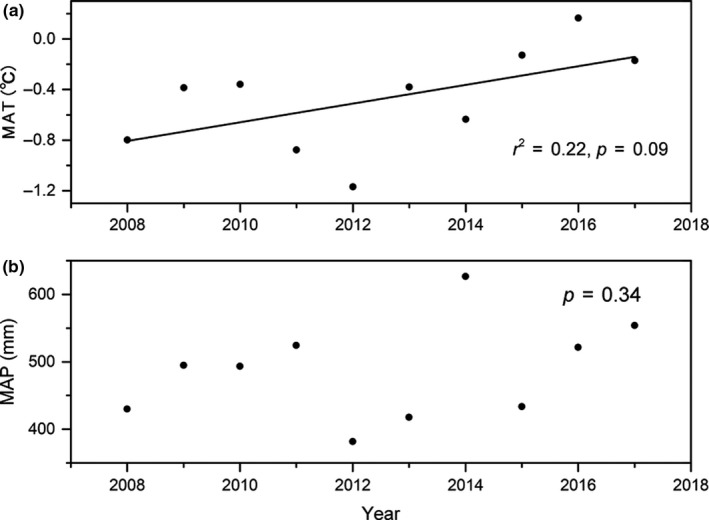
Interannual variation of climate variation. Note: MAT represents mean annual temperature; MAP presents mean annual precipitation. The same below

The mean annual temperature followed a weakly rising trend from 2008 to 2017 (*p* = 0.09; Figure 1a), with an averaged 10‐year mean temperature of −0.47°C, which increased at the rate of 0.7°C per decade. However, the mean annual precipitation did not show any significant change from 2008 to 2017 (*p* = 0.34; Figure 1b), when compared with the average 10‐year mean annual precipitation of 487.67 mm.

### 3.2 Interannual variations of AGB and BGB within the two vegetation types

**Figure 2:**
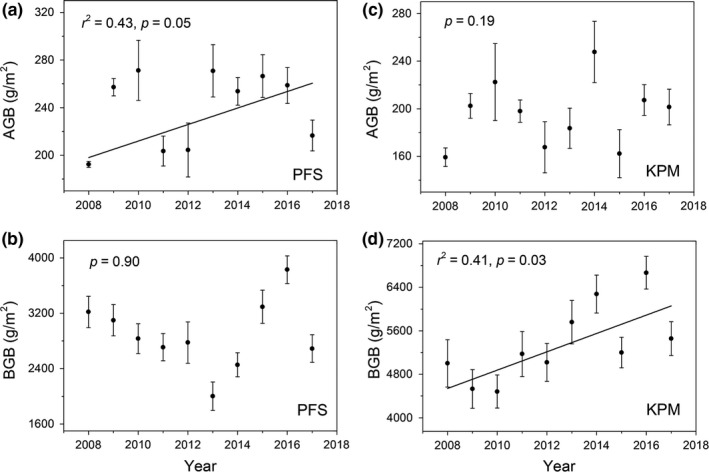
Interannual variation of aboveground biomass (AGB) and belowground biomass (BGB) in PFS and KPM. Note: PFS represents *Potentilla fruticosa* shrubs, KPM represents *Kobresia pygmaea* meadow, the same below

**Figure 3:**
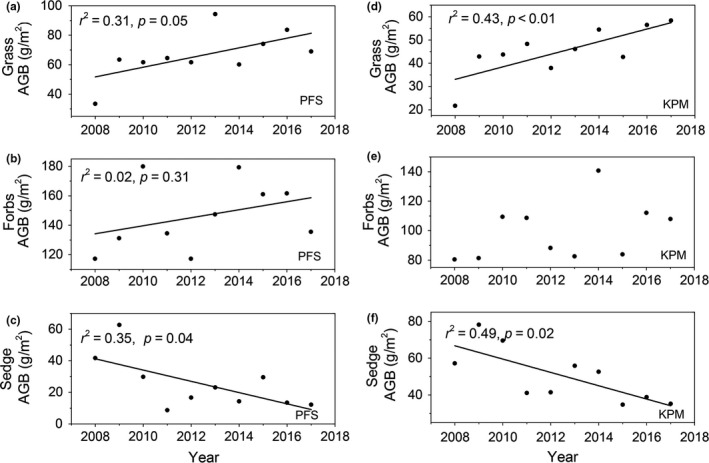
Interannual variation of aboveground biomass (ABB) among three functional groups in PFS and KPM

The AGB increased significantly from 2008 to 2017 in PFS (*p* = 0.05; Figure 2a), but the BGB showed no significant trend (Figure 2b). In contrast, the AGB displayed no significant change from 2008 to 2016 in KPM (Figure 2c), while the BGB increased significantly (*p* = 0.03; Figure 2d). Furthermore, the functional groups in plants responded differently to the enhanced temperature; the grass AGB in KPM and PFS showed a significant increasing trend from 2008 to 2017 (*p* < 0.01 and *p* = 0.05, respectively) (Figure 3a and d), the forbs AGB showed no significant change (Figure 3b and e), while the sedge AGB in KPM and PFS decreased significantly from 2008 to 2017(*p* = 0.02 and *p* = 0.04, respectively) (Figure 3f and c).

**Figure 4:**
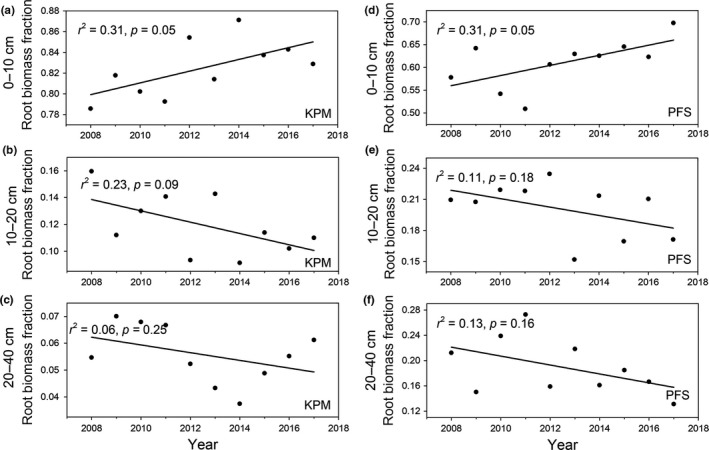
Interannual variation of root biomass fraction across different soil layers in PFS and KPM

In addition, the ratios of root biomass at 0–10 cm depth to total root biomass in KPM and PFS increased significantly (Figure 4a and d), while the ratios for the other two depth categories decreased (Figure 4b,c,e and f), reflecting a shift in biomass toward the surface layer.

### 3.3 Relationship between biomass and climate factors within the two vegetation types

**Figure 5:**
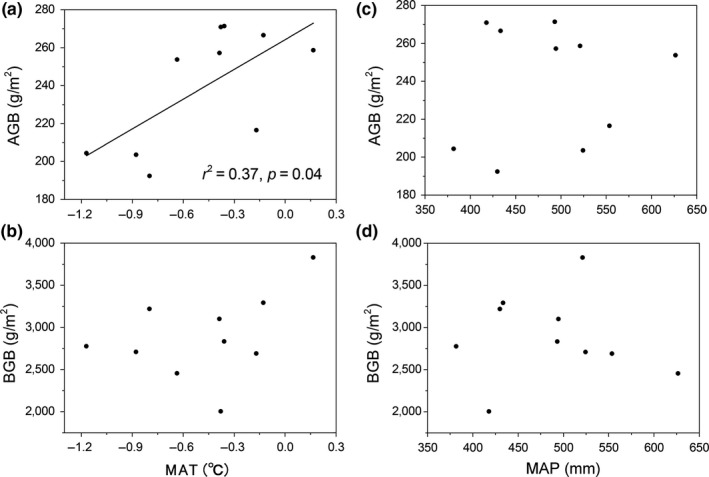
Relationship between aboveground biomass (AGB), belowground biomass (BGB), and climate variables in PFS

**Figure 6:**
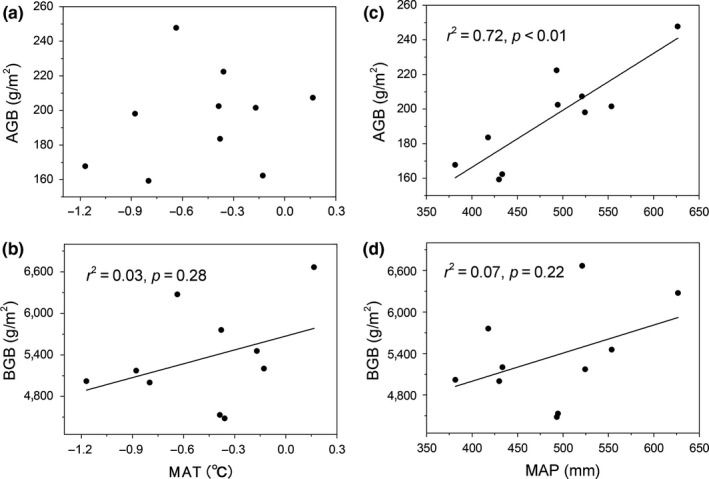
Relationship between aboveground biomass (AGB), belowground biomass (BGB), and climate variables in KPM

Regression analysis indicated that the PFS AGB was significantly positively correlated with MAT (Figure 5a), whereas the MAP exerted little impact on PFS AGB (Figure 5c). In contrast, the AGB of KPM was highly influenced by MAP rather than by MAT (Figure 6a and c). Furthermore, both MAT and MAP showed no significant impact on the BGB of PFS and KPM (Figures 5 and 6) and the interaction between MAP and MAT exerted no significant effect on either AGB or BGB across two vegetation types (*p* > 0.05; Table 2).

**Table 2:** Effects of climate factors on AGB and BGB among two vegetation types

		AGB			BGB		
Vegetation type	Climate factors	*df*	*F*	*p*	*df*	*F*	*p*
PFS	MAT	1	5.98	0.05	1	1.36	0.29
	MAP	1	0.03	0.87	1	0.22	0.66
	MAT × MAP	1	1.57	0.26	1	0.83	0.40
KPM	MAT	1	1.19	0.32	1	1.19	0.32
	MAP	1	17.14	0.01	1	0.93	0.37
	MAT × MAP	1	0.01	0.92	1	0.27	0.62

### 3.4 Root distribution and its seasonal dynamics within the two vegetation types

Based on asymptotic modeling of the vertical root distribution (7a and c), the *β* values for KPM and PFS were 0.84 and 0.92, respectively, reflecting the alpine shrubs have a deeper root distribution than alpine meadow classes. Moreover, the KPM had more root biomass (82%) distributed in the top soil layer (0–10 cm) than that of PFS (79%) (7b and d).

The seasonal variation of root fraction across different soil layers within the two root biomass patterns was relatively stable (Figure 8). Specifically, the 0–10 cm root fraction in KPM and PFS with only a slight decline from August to September, with decreases of 5% and 6%, respectively. In contrast, the 10–20, 20–30, and 30–40 cm root fractions increased by 21.22%, 31.81%, and 51.01% in KPM, and increased by 5.3%, 11.03%, and 11.51% in PFS, respectively.

**Figure 7:**
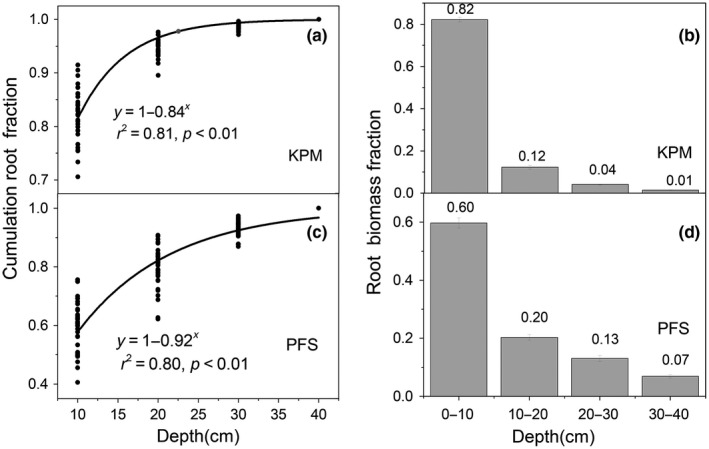
Vertical distributions of roots in KPM and PFS

**Figure 8:**
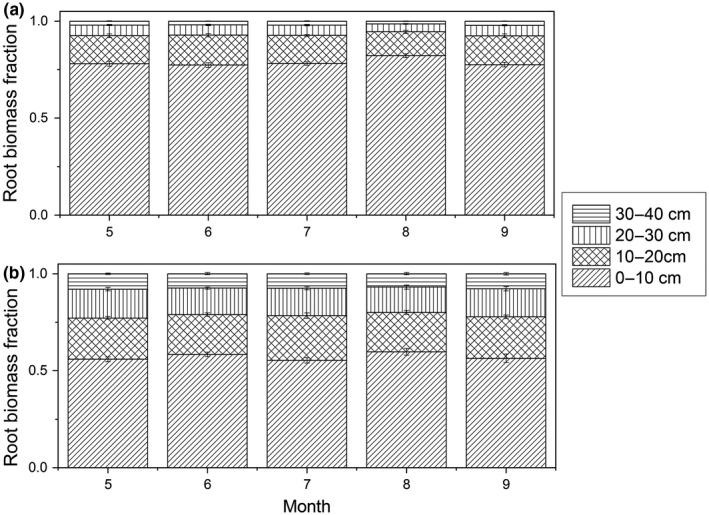
Seasonal variation in root fraction across different soil layers in KPM (a) and PFS (b)

### 3.5 AGB, BGB, and R/S within two vegetation types

The sizes of AGB, BGB, and R/S were greatly changed in both KPM and PFS. The AGB, BGB, and R/S in KPM ranged from 60.82 to 352 g/m^2^, 1642.63–11527.34 g/m^2^, and 11.94–75.84, respectively (Table 3). In PFS, the AGB, BGB, and R/S ranged from 103.63 to 539.20 g/m^2^, 249.21–6868.40 g/m^2^, and 4.95–40.14, respectively (Table 3). The AGB, BGB, and R/S differed significantly within the two vegetation types. The median values of AGB, BGB, and R/S in KPM were 191.22 g/m^2^, 5,181.30 g/m^2^, and 31.60, respectively, and those in PFS were 246.40 g/m^2^, 2,546.18 g/m^2^, and 13.15, respectively (Table 3). Overall, AGB in PFS was significantly higher than KPM, while the BGB and R/S in KPM were significantly higher than PFS (Table 3).

**Table 3:** The median values of aboveground biomass (AGB), belowground biomass (BGB) and BGB: AGB ratio (R/S) for two vegetation types

	AGB (g/m^2^)		BGB (g/m^2^)		R/S	
Vegetation type	Median	Range	Median	Range	Median	Range
KPM	191.22^b^	60.82–352.00	5181.30^a^	1642.63–11527.34	31.60^a^	11.94–75.84
PFS	246.40^a^	103.63–539.20	2546.18^b^	249.21–6868.40	13.15^b^	4.95–40.14

Note

Different letters indicate significant differences between two vegetation types.

## 4 DISCUSSION

### 4.1 Interannual variation and controls of biomass across two vegetation types

Our results indicated that the AGB of PFS showed a significant increasing trend from 2008 to 2017, while there was no significant trend in AGB of KPM (Figure 2), which might be attributed to the different responses of functional groups to climate change between the two vegetation types. For instance, the grass AGB increased significantly under enhancing temperature among two vegetation types. However, the sedges AGB in two vegetation types decreased significantly (*p* < 0.05). This evidence is also observed in a previous study at the same site which found that enhanced temperature increased grass relative abundance but reduced sedge relative abundance (Li, Zhang, Li, Zhao, & Cao, 2016; Liu et al., 2018). Thus, the significant increase in total AGB in PFS might be attributed to the increase in AGB of grasses and forbs surpassing the decrease in the AGB of sedges. Similarly, the lack systematic change in KPM AGB could stem from the significantly increase in grass AGB offseting the significantly decrease in sedges AGB.

In addition, the relationships between climate variables and biomass among the two vegetation types were also explored, revealing varied responses of biomass in different vegetation types to climatic variables. For instance, the AGB of KPM was more strongly influenced by MAP than by MAT (Figure 6), while the AGB of PFS was more strongly influenced by MAT than by MAP (Figure 5). This result is inconsistent with previous studies, in which the productivity of alpine ecosystem was limited mainly by low temperature rather than by precipitation (Sun et al., 2013). Therefore, we advise caution when exploring the relationships between biomass and climate variables due to the different responses of different vegetation types to climate change. Overall, temperature affects the total AGB by altering the biomass of functional groups; thus, the weak impact of temperature on KPM AGB might result from the balance effects at the functional group level (Bai, Han, Wu, Chen, & Li, 2004): that is, the positive effect of temperature on grass biomass offsets the negative effect of temperature on sedges biomass, ultimately leading to no significant impact of temperature on the total AGB in KPM. An alternative explanation for the discrepancy may link to the unique biological characteristics in KPM, that is, thick Mattic Epipedon (dense organic‐rich turf), which has a warming effect that can alleviate the stress of low temperature on plant growth, thus the plant in KPM may more limited by precipitation compared with temperature. In contrast, the BGB of both the vegetation types was affected less by climate variables, which did not agree with the results of a previous study that reported a decrease in belowground biomass with increasing temperature brought about by the reduction in soil moisture and increase in respiration (Shaver, Chapin, & Gartner, 1986). These discrepancies may be ascribed to the difference in climate and species composition. For instance, the thawing of seasonal freeze could alleviate the water stress to some extent during the growing season. Therefore, we suggested that the BGB in alpine meadow may be more affected by factors other than climate variation.

### 4.2 Vertical distribution of roots among two vegetation types

The root biomass was decreased with depth across two vegetation types, which could be roughly characterized by a "T" shape from shallow to deep soil layers, this result was consistent with previous study (Jackson et al., 1996). This unique root distribution feature might be partly caused by the soil nutrient distribution in which more soil nutrient are concentrated in the surface layers of the soil profile (Table 2); thus, the plant trends to allocate more biomass in the surface soil layers to absorb more nutrients. Furthermore, roots are more likely to near the surface of the soil to obtain more oxygen (Schenk & Jackson, 2002). Meanwhile, our results indicate that the alpine meadow root distribution in this study was shallower than that of the globally averaged root distribution for alpine grasslands (*β* = 0.97 and 0.93, respectively) (Jackson et al., 1996). These discrepancies could partially stem from different species compositions (Ma et al., 2017) and harsh climate conditions, such as seasonally frozen ground. Considering that seasonally frozen ground is well developed in this region, this may inhibit the root growth (Jackson et al., 1996). Furthermore, the different vegetation types may also greatly influence the vertical distribution of roots. In this study, compared with the global root distribution (including desert grassland, temperate grassland, and tundra), we have only explored the vertical root distribution of alpine meadows and shrubs; thus, the species composition is different, ultimately leading to the major discrepancies in vertical root distribution between our study and previous studies. In addition, we found that the KPM exhibited shallower root distributions, with 80% of root biomass concentrated in the top 10 cm of soil compared with the PFS allocate only 60% of roots located in the top 10 cm of soil (Figure 7).

Furthermore, we found a shift in biomass toward the superficial layer across the two vegetation types, this result was not consistent with the result of a previous study conducted in an alpine meadow (Wu et al., 2014). In general, root distribution was strongly associated with water availability and nutrient supply. As the climate in alpine meadows is relatively humid, the soil water content in the soil surface is usually abundant (Cao et al., 2004; Li et al., 2004). However, enhanced temperature could significantly decrease the soil moisture across different depths, especially at a depth of 10 cm (Liu et al., 2018). Thus, the plants tend to allocate more root biomass to shallower soil layers to obtain more moisture according to the optimal partitioning hypothesis that states the preferential allocation of more biomass by plants to parts with restricted growth to enhance their growth (McCarthy & Enquist, 2007; Skarpaas et al., 2016). Furthermore, another study found that both the rates of diffusion of nutrients to roots and nutrient availability could alter nutrient supply in plants further affecting root biomass distribution (Björk, Majdi, Klemedtsson, Lewis‐Jonsson, & Molau, 2007), and the enhanced temperature could affect nutrient supply by affecting the soil microbial community. For example, Zhang et al.(2014) observed that the 0–10 cm soil layer microbial biomass was increased significantly by warming, leading to a higher mineralization rate of *N* (Rustad et al., 2001). Considering that alpine ecosystems are nutrient poor, and that the soil nutrient supply is strongly influenced by nutrient mineralization during microbial decomposition, it is possible that the roots may develop greater biomass in the superficial layer as a strategy to obtain more soil nutrients. Overall, in our study, the warming magnitude in natural ecosystem during 2008–2017 was not obvious compared with those artificial warming control experiment. Therefore, a comparison experiment between natural warming and artificial warming would be conducted to have a better understanding how root distribution response to climate warming. Moreover, the control factors of vertical distribution of roots were quite complicated and affected by many factors such as climate variable, soil variable, and species composition; thus, more control factors should be considered in any future studies.

### 4.3 AGB, BGB and R/S among the two vegetation types

The AGB in PFS was significantly higher than KPM, while the BGB and R/S in KPM were significantly higher than PFS (Table 3), suggesting that more photosynthetic product was allocated to underground part in KPM compared with PFS, which may also reflect a unique survival strategy for alpine meadow plant to adapt the low temperature and shorter growing season. Some studies indicate that the existence of a shrub layer can provide a beneficial environment by increasing the soil and permafrost temperature (Myerssmith et al., 2011; Nie et al., 2017). Thus, compared with alpine shrubs, a slower consumption of energy and carbohydrates in the roots and lower turnover exist in alpine meadows because of the cold climate conditions. Compared with alpine meadows, the larger amount of litter, not only from woody plants but also from herbs in the shrub ecosystem (Nie et al., 2017, 2019), contributes to the accumulation of nutrition in the shrub ecosystem. Combining these factors, the roots in alpine meadows may be likely to allocate more photosynthates to the roots to absorb more nutrition. Overall, the median values of AGB, BGB, and R/S in KPM were higher the mean of China's grasslands (Yang et al., 2010), and also higher than those of global grasslands (Jackson et al., 1996). These discrepancies might be partially attributed to the climate differences: specifically, plants in the alpine meadow ecosystem were mainly limited by low temperatures, less precipitation, and poor nutrient conditions. In general, plants may be likely to allocate more photosynthates to roots in poor nutrient and low temperature, but shift more photosynthates to shoots in good nutrient conditions according to the functional equilibrium hypothesis (optimal partitioning) (Sun & Wang, 2016). Therefore, the combination of low temperature and poor nutrient conditions lead to a higher biomass allocation to the roots, ultimately resulting in a higher R/S in the alpine ecosystem than in other regions. Meanwhile, the larger R/S ratio in the alpine meadow could be partially attributed to the relatively slow consumption of energy and carbohydrates in roots and lower root turnover because of cold climate condition (Davidson, 1969; Gill & Jackson, 2000).

Furthermore, a higher R/S was observed in alpine shrubs than the median R/S of global shrubs (1.84) (Mokany, Raison, & Prokushkin, 2006), which might be caused by the shorter growing season in alpine shrubs, with more photosynthetic products allocated to belowground parts, resulting in a larger R/S in alpine shrubs. Previous studies have found that the allocation of plant biomass varied with ecosystem and functional groups: for example, the plants in alpine meadow tend to allocate more biomass to roots (Wu et al., 2011), whereas forbs might allocate more biomass to shoots in tundra ecosystems.

## 5 CONCLUSIONS

This study aimed to explore the interannual variations of plant biomass within two vegetation types and their relationships with climate variables on the Qinghai–Tibetan Plateau, based on long‐term observations. We found that the responses of plants to climate change varied between vegetation types. MAT exerted a significant influence on AGB in PFS, but had no significant impact on AGB in KPM. Instead, the AGB in KPM was dominated by MAP. Furthermore, the BGB in both KPM and PFS was only weakly affected by climate variables, indicating that the BGB in alpine ecosystems may remain as a stable carbon stock in the future. Furthermore, the proportion of 0–10 cm root biomass increased among two vegetation types under climate fluctuations, whereas root biomass in the other proportions decreased, reflecting a shift in biomass toward the superficial layer and demonstrating the unique survival strategies of alpine plants. Our results indicated that biomass allocation varied between vegetation types under current climate fluctuations and the root biomass induced by elevated temperature tends to allocate more biomass to the surface soil layer, thereby providing new insights into the response of alpine ecosystems to climate change.

## CONFLICT OF INTEREST

None declared.

## AUTHOR CONTRIBUTION

L Dai performed the research, analyzed data, and wrote the paper; F Zhang, X Guo, X Ke, Y Li, Y Du, C Peng, L Lin, Q Li and K Shu analyzed data; G Cao conceived the study.

## DATA AVAILABILITY

The biomass and climate data are available in Dryad: Dryad https:// https://doi.org/10.5061/dryad.2t14h9r.
